# Mapping evidence on standards and quality of care for older persons in long-term care facilities: a scoping review protocol

**DOI:** 10.1186/s13643-021-01709-2

**Published:** 2021-05-22

**Authors:** Letasha Kalideen, Pragashnie Govender, Jacqueline Marina van Wyk, Desmond Kuupiel

**Affiliations:** 1 School of Clinical Medicine, College of Health Sciences, University of KwaZulu-Natal, Private Bag X54001, Durban, 4000 South Africa; 2 School of Health Sciences, College of Health Sciences, University of KwaZulu-Natal, Private Bag X54001, Durban, 4000 South Africa; 3 Centre for Evidence-based Health Care, Department of Global Health, University of Stellenbosch, Stellenbosch, 7602 South Africa; 4 Research for Sustainable Development Consult, Sunyani, Ghana

**Keywords:** Standards of care, Long-term care facilities, Elder care, Quality health care, Healthy ageing

## Abstract

**Background:**

Ageing is a global and universal process that results in physiological, psychological and behavioural changes. Due to the changing needs of the individual and the circumstances of the family, long-term care of older persons in facilities has become essential. The standard and quality of health care for older persons in long-term care facilities is critical to maintain functional ability in keeping with international goals of healthy ageing. This study, therefore, will aim to systematically map literature and describe the evidence on standards and the quality of health care for older persons living in long-term care facilities (LTCFs).

**Methods:**

A scoping review will be conducted using Arksey and O’Malley’s framework and recommendations set out by Levac and colleagues. PubMed, CINAHL, Health Sources, PsycInfo, Web of Science, Scopus, and Google Scholar will be searched for relevant published studies/sources of evidence up to the last search date. The search will be conducted using keywords, and Boolean terms, and Medical Subject Headings/Subject Headings. EndNote X9 will be used to compile all relevant sources of evidence. This study will include studies involving participants ≥ 65 years old, living in LTCFs for older persons. English language publications, with no time limitations, and primary studies, guidelines, and quality of care specific to LTCFs for older persons will be sourced. Two reviewers will independently screen all sources of evidence at the title, abstract, and full-text screening stages as well as perform the data charting. The Preferred Reporting Items for Systematic Review and Meta-Analysis flow diagram will be used to account for all relevant sources of evidence during the screening. Thematic content analysis will be used to describe the themes aligned with this study’s research question based on initial coding and categorisation and a summary of the findings reported narratively for each theme.

**Discussion:**

We anticipate that this scoping review will highlight the standards of care and assessment tools available worldwide, in addition to gaps that exist in the evidence base for older persons in LTCFs. These may include an exposition of the standards and quality of care documented, monitoring and evaluation processes, instruments used for reviewing standards of care. This would contribute towards identifying the need for practical and universally acceptable tools for LTCFs for older persons.

## Background

Ageing is a global and universal process that results in physiological, psychological and behavioural changes. In 2019, there were 703 million persons aged 65 years or over globally, indicating an increase from 6% in 1990 to 9% in 2019 [1]. Globally, it is further projected that the number of persons aged 65 years or over will double to about 1.5 billion in 2050 (about 16% rise by 2050) [1]. In sub-Saharan Africa, the proportion of older persons estimated at 43 million in 2010 is projected to reach 67 million by 2025 and 163 million by 2050 [2]. Ageing is not a disease, but it increases the propensity for diseases such as hypertension, diabetes and cardiovascular disease due to a decline in functional capacity [2, 3]. Hence, long-term care of older persons is often necessary due to the changing needs of the individual and the social circumstances of the family [4].

Long-term care, according to the World Health Organisation (WHO), include “activities undertaken by others to ensure that people with or at risk of a significant on-going loss of intrinsic capacity can maintain a level of functional ability consistent with their basic rights, fundamental freedoms and human dignity” [5]. This definition aims to focus on the quality of life, including nutrition, rehabilitation, and physical activity. In its global strategy and action plan on ageing and health, the WHO reaffirms the promotion of healthy ageing and emphasise the development and maintenance of functional ability that enables well-being in older age [6]. Similarly, the United Nations 2030 agenda for the Sustainable Development Goals (SDG) Plan of Action has identified the need to protect and promote the rights of the older person [7]. Older persons are not only at high risk of diseases but also at high risk of dying from chronic diseases if not well manage according to prescribed standards or guidelines [8–11].

To this end, compliance to clinical standards such as clinical practice guidelines, health care protocols, standard procedures, quality improvement assessment tools, legal framework and others non-clinical standards are essential to help improve the standard and quality of health care rendered to older persons, particularly those in LTCFs. Although several previous research studies have aimed to promote the quality of care rendered to older persons [12–18], literature gaps may still exist requiring further research to help inform policy. Several review studies have also been previously conducted [14, 19, 20], but to date, no study has systematically mapped literature on standards and the quality of health care provided to older persons living in LTCFs worldwide hence, informing this study.

This current study, therefore, will aim to systematically map literature and describe the evidence on the standards and quality of care for older persons living in LTCFs globally. This study will also aim to identify literature gaps around standard guidelines for the care of older persons and make recommendations for practitioners, policy-makers and researchers towards quality improvement of the standard of care rendered to older persons living in LTCFs. Quality improvement science is an emerging field that is associated with other areas of research like implementation science, translational science, health care delivery science and knowledge translation [21]. The focus towards quality improvement science is to transform research into common practice to improve care processes and outcomes [21]. Hence, a study such as this is essential to improve the standard of care in LTCFs.

## Methods

A scoping review would be conducted guided by the Arksey and O’Malley framework incorporating Levac and colleagues recommendations [22, 23]. A scoping study approach enables a systematic search for literature, examination and selection of literature, knowledge synthesis, and mapping of concepts and the range of evidence to address an exploratory research question [22, 24]. In addition, a scoping review study enables the authors to identify gaps in research for future investigation [22]. The Preferred Reporting Items for Systematic Review and Meta-Analysis extension for protocols (PRISMA-P) was adopted to develop this protocol but, the PRISMA extension for a scoping review (PRISMA-ScR) checklist will be followed to report this study [25]. This study will use the following steps outlined in the Arksey and O’Malley framework [22]:

1. Identifying the research question
2. Identifying relevant studies
3. Study selection
4. Charting the data
5. Collating, summarising and reporting the results

### Identifying the research question

This scoping review will seek to answer the following question: *“To date, what evidence exists on standard and quality of care for older persons living in LTCF globally?”* The sub-questions for this review will include the following:

1. What standards of care, including assessment tools, exist for the care of older persons living in LTCF?
2. How are the existing standards meeting the needs (quality of care) of older persons living in LTCF?
3. What are the gaps in the literature about the standard and quality of care for older persons living in LTCFs?

The population, concept and context (PCC) acronym were used to define the research for this study (Table 1).

**Table 1:** PCC acronym used to define the eligibility of the primary research question for this review

P: Population	Elderly: This includes individuals aged ≥ 65 years
C: Concept	Standard of care: this will include clinical standards such as clinical practice guidelines, health care protocols, standard procedures, legal frameworks, quality improvement assessment/appraisal tools for assessing the quality of care for older persons
C: Context	Quality of care: this study will adopt the WHO definition of quality of care which states that “the extent to which health care services provided to individuals and patient populations improve desired health outcomes. To achieve this, health care must be safe, effective, timely, efficient, equitable and people-centred.” [26]

### Identifying relevant studies/evidence

To answer this review question, the authors, with support from a subject-librarian, will conduct a systematic search in the following electronic databases: PubMed, CINAHL, Health Sources, PsycInfo, Web of Science, Scopus, and Google Scholar for relevant published studies from the inception date of the database to the last search date. This study will use a complete search strategy that employs keywords, medical subject headings (MeSH) or subject headings search terms that relate to key concepts, as well as Boolean terms “AND” and “OR”. Year of publication and study design and language limitations will be removed during the search to enhance the capturing of all possible relevant articles. Each search record will be appropriately documented as follows: date of search, database, keywords, number of retrievable studies, and number of eligible studies. A search strategy piloted in PubMed is presented in Table 2. The subject-librarian will assist in optimising the search strategy within the electronic databases. The electronic search strategy will also be guided by the Peer Review of Electronic Search Strategies (PRESS) statement [27]. This study will also source grey literature such as guidelines, policies, theses and dissertations, and other articles relevant to answer the research question using Google search engine. Moreover, relevant studies or grey literature from the reference list of the included studies will be sourced using a snowball approach. EndNote X9 reference manager will be used to compile all relevant studies/grey and identify duplicate records for removal.

**Table 2:** Pilot search in PubMed electronic database

Date	Keywords	Search results
12/05/2020	(((((((((clinical[All Fields] AND standard[All Fields]) OR (“practice guideline”[All Fields] OR “practice guidelines as topic”[MeSH Terms] OR “clinical practice guideline”[All Fields])) OR ((“delivery of health care”[MeSH Terms] OR (“delivery”[All Fields] AND “health”[All Fields] AND “care”[All Fields]) OR “delivery of health care”[All Fields] OR (“health”[All Fields] AND “care”[All Fields]) OR “health care”[All Fields]) AND protocol[All Fields])) OR (standard[All Fields] AND (“methods”[MeSH Terms] OR “methods”[All Fields] OR “procedure”[All Fields]))) OR (legal[All Fields] AND framework[All Fields])) OR (((“quality improvement”[MeSH Terms] OR (“quality”[All Fields] AND “improvement”[All Fields]) OR “quality improvement”[All Fields]) AND “assessment”[All Fields]) OR (appraisal[All Fields] AND tool[All Fields]))) OR (“standard of care”[MeSH Terms] OR (“standard”[All Fields] AND “care”[All Fields]) OR “standard of care”[All Fields])) AND (“quality of health care”[MeSH Terms] OR (“quality”[All Fields] AND “health”[All Fields] AND “care”[All Fields]) OR “quality of health care”[All Fields] OR (“quality”[All Fields] AND “care”[All Fields]) OR “quality of care”[All Fields])) AND ((“aged”[MeSH Terms] OR “aged”[All Fields] OR “elderly”[All Fields]) OR (“aged”[MeSH Terms] OR “aged”[All Fields]) OR (old[All Fields] AND (“persons”[MeSH Terms] OR “persons”[All Fields] OR “people”[All Fields])) OR geriatric[All Fields])) AND ((“nursing homes”[MeSH Terms] OR (“nursing”[All Fields] AND “homes”[All Fields]) OR “nursing homes”[All Fields] OR (“long”[All Fields] AND “term”[All Fields] AND “care”[All Fields] AND “facility”[All Fields]) OR “long term care facility”[All Fields]) OR facilities[All Fields])	13,227

### Eligibility criteria and selection of studies/evidence sources

#### Eligibility criteria

This proposed study’s eligibility criterion is summarised below (Table 3)

**Table 3:** Eligibility criteria for selection of evidence/studies

Eligibility criteria	Inclusion criteria	Exclusion criteria
Source of evidence	Electronic database, reference list of included studies and grey literature existing in Google website	
Population	Individuals ≥ 65 years old	
Concept	Standard of care (clinical practice guidelines, health care protocols, standard procedures, legal frameworks, quality improvement assessment/appraisal tools	
Context	Quality of care in LTCF such as Nursing homes, and long-term rehabilitation facilities	Hospitals, mental health facilities, and sub-acute and acute facilities
Publication status	Published peer-reviewed studies, and grey literature	
Language	English	Publications/grey literature in other languages such as French, Arabic, Chinese, and others
Study designs	Primary study designs such as quantitative, qualitative, and mix-method studies with human participants as well as guidelines	Other review studies such as systematic, literature and scoping reviews

#### Study/evidence source selection

**Fig. 1:**
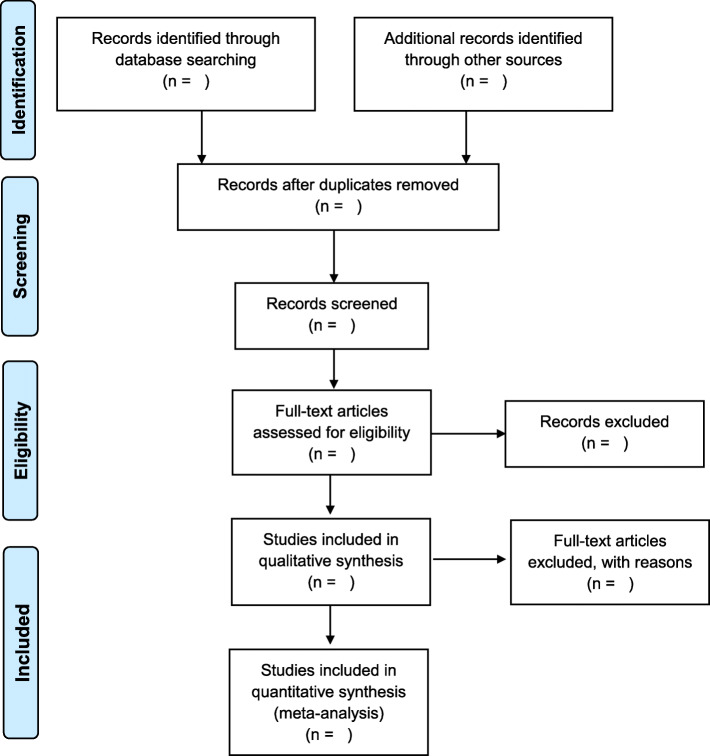
PRISMA 2009 Flow Diagram [28]

Prior to the selection of evidence, all the methods (title, abstract and full-text screening) will be pilot tested to calibrate operators and increase consistency and to fine-tune the methods. LK and DK will used hundred titles, and ten per cent each of the sources of evidence included at the abstract and full-text stage for the pilot testing. LK and DK will conduct a thorough title screening of the electronic databases guided by the eligibility criteria. All relevant articles will be imported into an Endnote library and duplicates removed. Following this the EndNote library will be shared amongst the review team for the next stage of the study selection process. PG and JvW will verify to ensure that all relevant titles have been captured prior to abstract screening. A screening tool will be developed in Google Forms using the eligibility criteria for the abstract and full-text screening phases. Two reviewers (LK and DK) will independently conduct abstract and full-text screening phases and group them into either an “include” or “exclude” category. Discrepancies between LK and DK at the abstract screening phase will be addressed through a discussion by the review team until a consensus is reached. At the full-text phase, JvW will resolve any discrepancies between LK and DK. Where an article could not be accessed freely online, assistance from the institution’s library services will be sought. The original authors will also be accessed via email for requests of full texts, if necessary. Cohen’s kappa coefficient (κ) statistic will be calculated to determine the inter-rater agreement between the reviewers at the end of the full-text screening phase. The PRISMA flow diagram will be adopted to report the screening results [28] as illustrated in Fig. 1 below.

### Charting the data

A form will be developed in Google Forms for the data extraction and piloted with 10% of the included sources of evidence to ensure its accuracy. LK and DK will independently extract all relevant data from the included articles after a thorough reading of the full texts. A hybrid approach using both inductive and deductive approaches will be used to extract the data from the included studies [29]. The data extraction form will include the following details, namely, (1) author and year of publication, (2) title of evidence/study, (3) aims and objective, (4) country of the evidence/study, (5) study design, (6) study participants, (7) study results, (8) findings relevant to answer the question, (9) conclusion and (10) recommendations. The form will be continually updated to enable the capturing of all relevant data to answer the review question.

### Quality appraisal

Although critical appraisal of individual sources of evidence is not mandatory for scoping reviews, this study will include this step to assess the methodological quality of primary studies. This will enable to determine if the conclusions drawn and recommendations made on a type of standard and the quality of care in a LTCF was based on a valid method. To facilitate this, this study will use the mixed method quality assessment tool [30]. A prescribed set of questions for each study type will be employed in order to examine the appropriateness of the study objective/question, study design, participant recruitment, data collection, data analysis, and the results reported for each included study. The scores will be graded and a quality score of ≤ 50% will be interpreted as low quality, and a score ranging from 51 to 75% will be interpreted as average quality [31]. A score ranging from 76 to 100% will also be interpreted as high quality [31]. To reduce bias, two reviewers will independently conduct the quality appraisal.

### Collating, summarising and reporting the results

Thematic content analysis will be used to describe the themes aligned with this study’s research question based on initial coding and categorisation [29]. Relevant themes that describe the standards and quality of care for older persons living in LTCF will also be constructed. Due to the qualitative nature of scoping review studies, we may conduct a follow-up meta-analysis using the quantitative data from this study.

## Discussion

The scoping review will provide valuable information on the current evidence on the standards and quality of care for LTCFs. This will inevitably assist in developing strategies that seek to focus on improving the quality of life, facilitate healthy ageing and maintaining the functional ability amongst older persons in LTCF [6]. The available resources and assessment tools available worldwide will also expose gaps in the availability, relevance and sensitivity of instruments for the standard and quality of care in LTCFs. This may then elucidate future studies on practical and universally acceptable tools for the LTCFs. This scoping review will be limited to publications in English due to a lack of expertise in other international languages. Due to funding constraints, it will further be limited to LTCFs for older people. All other limitations will be adequately reported in the results manuscript. Nonetheless, the finding of this study will be disseminated through conferences, key stakeholder meetings, and peer-reviewed publications.

## Data Availability

N/A.

## Abbreviations

LTCF: Long-term care facilities
WHO: World Health Organisation
